# Is Stress Relaxation in Sea Cucumber Dermis Chemoelastic?

**DOI:** 10.3390/md21120610

**Published:** 2023-11-25

**Authors:** Ettore Barbieri, Himadri Shikhar Gupta

**Affiliations:** 1 Center for Mathematical Science and Advanced Technology (MAT), Research Institute for Value-Added-Information Generation (VAiG), Japan Agency for Marine-Earth Science and Technology (JAMSTEC), Yokohama 236-0001, Japan; 2 School of Engineering and Materials Science (SEMS) & Institute of Bioengineering (IOB), Queen Mary University of London, London E1 4NS, UK

**Keywords:** chemoelasticity, sea cucumber, finite element method, first-order reactions

## Abstract

Echinoderms, such as sea cucumbers, have the remarkable property of changing the stiffness of their dermis according to the surrounding chemical environments. When sea cucumber dermal specimens are constantly strained, stress decays exponentially with time. Such stress relaxation is a hallmark of visco-elastic mechanical behavior. In this paper, in contrast, we attempted to interpret stress relaxation from the chemoelasticity viewpoint. We used a finite element model for the microstructure of the sea cucumber dermis. We varied stiffness over time and framed such changes against the first-order reactions of the interfibrillar matrix. Within this hypothetical scenario, we found that stress relaxation would then occur primarily due to fast crosslink splitting between the chains and a much slower macro-chain scission, with characteristic reaction times compatible with relaxation times measured experimentally. A byproduct of the model is that the concentration of undamaged macro-chains in the softened state is low, less than 10%, which tallies with physical intuition. Although this study is far from being conclusive, we believe it opens an alternative route worthy of further investigation.

## 1. Introduction

Mutable collagenous tissue (MCT), found in all echinoderms, is a remarkable example of a collagenous tissue that can alter its mechanical state (e.g., stiffness) within a few seconds [1]. Echinoderms are an evolutionarily ancient animal phylum and include starfish, sea cucumbers, and sea urchins. Physiologically, mutability in MCT mechanics manifests in energy-efficient rigid posture maintenance with minimal muscle involvement [2], in irreversible softening processes, including autotomy (or defensive tissue detachment) [3], or in the generation of tensile force in feather stars [4]. MCT has attracted attention as a template for developing mechanically adaptive materials, with applications in biomedical, cosmetics, and bioinspired fields [1,5,6,7]. Understanding the dynamic mechanical and stress-relaxation behavior of MCT can have important implications in the biomedical materials and cosmetics field. The ability to regulate (increase or decrease) the stress relaxation rate in MCT (or its mimics) by controlling chemical bond breakage and reformation could enable the development of reconfigurable or injectable biomaterial implants in soft tissues. Especially in tissues with very low stiffness, such as neural tissue and its surroundings, regulating or increasing the stress relaxation or creep of MCT-based biomaterials would facilitate implantation without injury. In a similar manner, our understanding of the time dependence of mechanical properties in MCT would aid in optimizing the processes of the topical application and absorption of collagen-based cosmetics derived from MCT.

However, there is still a lack of clarity on how mechanical mutability—especially dynamic or time-dependent changes—occurs in MCT, specifically in models that link the extracellular matrix (ECM) to mechanical behavior.

### 1.1. Structure and Mechanics

Structurally, the major components of the MCT ECM are collagen fibrils with a characteristic axial electron-density periodicity *D* (≈65–67 nm) [1,8,9] and a heterotrimeric structure at the molecular level, along with fibrillin-rich microfibrils, and proteoglycans in the interfibrillar space. Neural effector cells (called juxtaligamental cells or JLCs), closely interpenetrating the ECM, are likely to play a significant role in modulating MCT mechanics by releasing effector molecules like tensilin or softenin [10,11], though the evidence is still indirect. Biomechanically, MCT stiffness (elastic modulus) ranges from 1–200 MPa on average [1], assessed using tensile tests or cyclic dynamic mechanical analysis. Failure strains of between 20–60% are intermediate between those of aligned fiber composites like tendons and tissues, with a wide range of fiber orientations like skin [1,12]. A change in mechanical properties can be induced in the laboratory by changing the ionic composition of the tissue bathing fluid or by mechanical stimulation. Regarding the modulus, elevations of 4–6*x* have been reported for immersion in potassium-enriched artificial seawater (KASW) [8] and a factor of 2–3*x* for mechanical stimulation [13,14]. A range of biological effector molecules isolated from MCT, like tensilin, NSF, softenin, and stiparin, have been implicated in the stiffening and softening of MCT [1], potentially by increasing interfibrillar crosslinking or stiffening the interfibrillar matrix. Local water content has also been known to change (reduce) during MCT stiffening [15], which can cause increased or closer fibril packing, which, in turn, may lead to greater interfibrillar stress transfer and stiffness. In this regard, fiber-composite models have been proposed to explain the variation in mechanics in MCT due to changes in the structure and properties of the ultrastructural constituents—the collagen fibrils and interfibrillar ECM components [8,16]. By using synchrotron X-ray nanomechanical imaging to quantify the fibril strains and orientation under different states of mechanical stimulation and quasi-static deformation, we showed that mutability in MCT arises from changes in the interfibrillar matrix properties and effective interfibrillar cohesion [8] rather than changes in collagen fibril properties.

### 1.2. Modeling the Time-Dependent Behavior

However, modeling the dynamic mechanical changes of MCT ECM—like stress relaxation, creep, or changes in mechanical state—in terms of the behavior of the ultrastructural building blocks is more challenging. MCT exhibits the visco-elastic behavior seen in most hydrated soft tissues, but the characteristics change with the altered mechanical state [17]. In order to estimate viscosity or flow in the ECM, earlier experiments tested the speed of bending of tissues under set weights and in the presence of stiffening factors [10] or with changes in calcium concentration [18], as the force is proportional to strain rate in Newtonian viscosity, the time for bending was taken as proportional to material viscosity. Structurally, however, the mechanisms by which the ECM mechanics change during typical time-dependent mechanical alterations, like stress relaxation, have been studied little at the ultrastructural level. Recently, we used experimental data on the time-dependent fibril stress-relaxation in chemically stimulated MCT (determined using small-angle X-ray scattering) to develop an ultrastructural model with shear-lag between the fully elastic collagen fibrils embedded in a viscous extra-fibrillar matrix, (which can contain fibrillin, proteoglycans, water, and effector molecules) [17].

In this previous work [17], we assumed a fully elastic behavior for the fibrils embedded into a visco-elastic interfibrillar matrix. A shear-lag model connected the fibrils and matrix. The matrix can only withstand shear stress, τ, and the relationship with the shear strain, γ, is related to a convolution integral with the derivative of the interfibrillar matrix shear modulus $G_{m}\left(t\right)$, which was unknown.

(1) $$\tau \left(t\right)=G_{m}\left(0\right)\;\gamma \left(t\right)-\int_{0}^{t}\;\frac{dG_{m}\left(t-\tau \right)}{d\tau }\;\gamma \left(\tau \right)\;d\tau$$

Equation (1) is typical of visco-elastic behaviors [19]. We then obtained $G_{m}\left(t\right)$ and tried to explain the origin of $G_{m}\left(t\right)$ visco-elasticity by framing the visco-elastic parameters within an extended Doi-Edwards model. This interpretation agreed with some intuitive findings on the visco-elasticity of sea cucumbers: the increase in crosslinks and interchain friction from a standard-to-stiffening solution and the decrease in crosslinks and interchain friction from a standard-to-softening solution. However, these two results implied an apparent counter-intuitive conclusion on the elastic chain scission of the interfibrillar matrix. Specifically, we found that the molecular weight decreased during stiffening, and it increased during softening.

In order to address this discrepancy, we interpret stress relaxation as a chemical process in this paper, following [20], where elastically active chains and crosslinks break and reform dynamically. Here, by elastically active chains, we mean the chains in tension contributing to the shear modulus, $G_{m}\left(t\right)$, as in the molecular theory of rubber elasticity [21].

(2) $$G_{m}\left(t\right)=N\left(t\right)\;k\;T$$

where *k* is the Boltzmann constant, *T* is the absolute temperature, and *N* is the number density of elastically active chains.

In a chemoelastic stress relaxation process, *N* changes over time due to chemical reactions. The chemoelasticity, which is sometimes referred to as chemorheology, has been applied, for example, to model aging-induced fracturing [22], a high-temperature elastomer response [23,24], and reversible crosslinking in hydrogels [25].

In summary, firstly, we built a staggered finite element numerical model. We extracted an approximate solution of the finite element model to obtain insights into the possible mechanisms behind tissue stress relaxation. From this solution, we inferred that a chemoelastic model with a shear modulus that degrades exponentially with time is very plausible. Based on this assumption, we used an optimization algorithm to derive the mechanical parameters. Finally, we explain these mechanical parameters for fibrils and interfibrillar matrix with first-order reaction kinetics to reveal the mechano-cracking mechanisms.

## 2. Materials and Methods

The experimental results, re-analyzed here using the chemoelastic model, were collected, reported, and published in previous papers [8,17]. Hence, only a summary of the experimental methods used in those papers is provided here as the experimental work was not part of the current study, and we refer to [8,17] for the details. Black sea cucumber *(Holothuria atria)* specimens were collected from a commercial wholesaler. After placing them in a $-20^{\circ }$ freezer, sections of tissue of $12\;\mathrm{mm}\times {\left(1.8\;\mathrm{mm}\right)}^{2}$ were dissected out. All studies were carried out in accordance with the Animals (Scientific Procedures) Act 1986 of the UK, including revision 2013: invertebrates (except cephalopods) are not considered protected species under the Act. Samples were incubated in artificial (ASW; control), potassium-enriched ASW (KASW), and calcium-free ASW (CaFASW) seawater, following the protocols published previously [8,26]. Synchrotron SAXS measurements were carried out at beamline I22, Diamond Light Source (Harwell, UK), using an X-ray energy of 12.46keV, a collimated beam size of $20\times 25\;\mu m^{2}$, and an exposure time of 0.5 s per SAXS pattern. For the SAXS measurements, the tissues were mounted in a hydrated condition in a custom micromechanical tester in line with the X-ray beam at BL-I22 (developed by us [8]), using sandpaper to ensure adhesion to the grips. Stress-relaxation tests were carried out as described, and the SAXS patterns were azimuthally integrated to obtain radial line plots, from which the fibril strain was calculated as a percentage change in the 5th-order Bragg peak from the meridional fibril scattering, as described previously [8]. By combining the stress, tissue strain, and fibril strain plots as functions of time, the tissue and fibril-level mechanical data re-analyzed in this paper were obtained.

**Figure 1:**
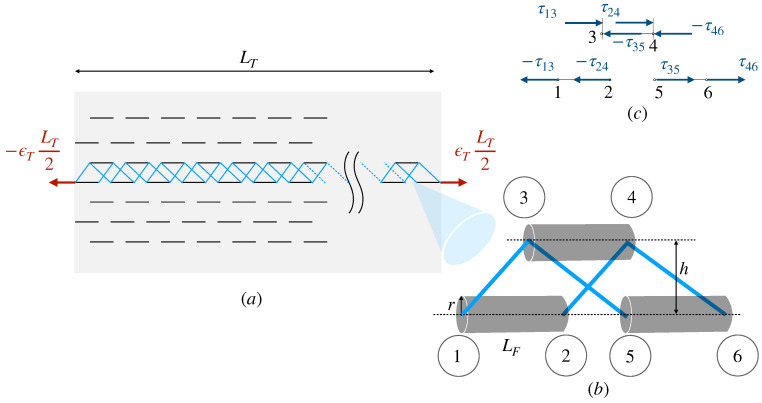
Finite element model of staggered fibrils of length $L_{F}$, connected by an interfibrillar matrix. (**a**) Tissue specimen of length $L_{T}$, with an applied constant strain $\epsilon_{T}$ and fibrillar microstructure; (**b**) fibril elements (cylinders), shear elements (blue lines) and node numbering (circled numbers); (**c**) The matrix cannot carry tensile loads, only shear stresses, $\tau_{ij}$.

We formulate a shear-lag finite element model, where the interfibrillar matrix can only transmit shear stresses but not carry tensile loads (Figure 1). The implementation and solution details are in Appendix A.

We proceed in two steps. Firstly, we use approximated analytical results (Appendix B) to deduce qualitatively the mechanical properties of the fibrils and matrix. Such an analytical solution can be obtained only for a small number of fibrils; for more fibrils, the models can only be solved numerically.

Secondly, we use full numerical simulations to quantitatively derive the parameters of the elastic models for fibril and matrix for all the cases.

From the qualitative approximate analytical solution (Appendix B), we obtain

(3) $$\sigma_{T}\left(t\right)\approx \frac{1}{2}\;N\;\rho_{F}^{2}\;\sqrt{\frac{\phi }{\pi }}\;G_{m}\left(t\right)\;\epsilon_{T}$$

where *N* is the number of fibrils, ϕ is the fibril volume fraction, $\rho_{F}$ is the fibril aspect ratio, and $\epsilon_{T}$ is the constant applied strain.

**Figure 2:**
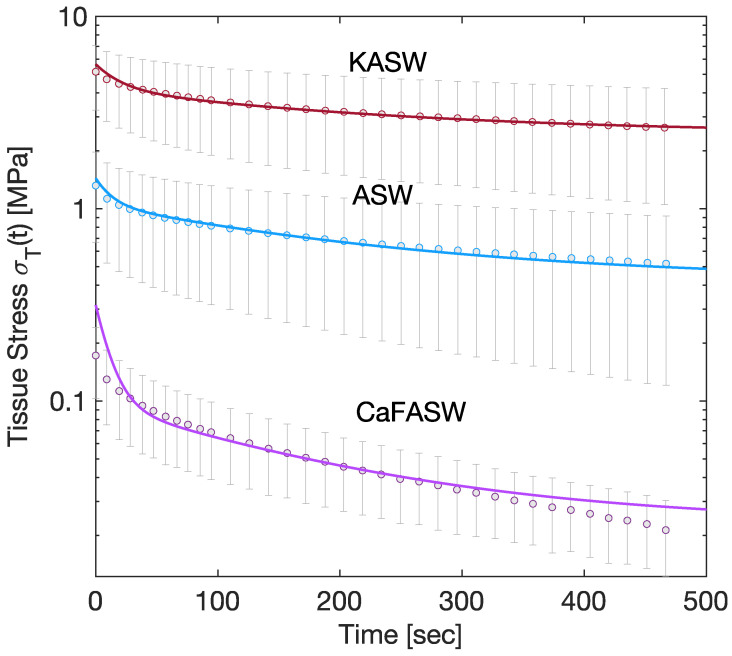
Chemoelastic relaxation for tissue stress; the continuous lines are the numerical results, the dots are the mean of the experimental measures, and the error bar plots are the corresponding standard deviations.

Equation (3) states that the tissue stress directly relates to the interfibrillar matrix. Experimentally, the tissue stress is captured by a two-term Prony series [8,17], as seen in Figure 2. Therefore, in the following, we assume that $G_{m}\left(t\right)$ is given by

(4) $$G_{m}\left(t\right)=G_{\infty }\;\left(1+\gamma_{1}\;e^{-t/t_{M_{1}}}+\gamma_{2}\;e^{-t/t_{M_{2}}}\right)$$

where $G_{\infty }$ is the asymptotic shear modulus, $\gamma_{1}$ and $\gamma_{2}$ are dimensionless parameters of magnitude O(1), and $t_{M_{1}}<t_{M_{2}}$ are time constants.

**Figure 3:**
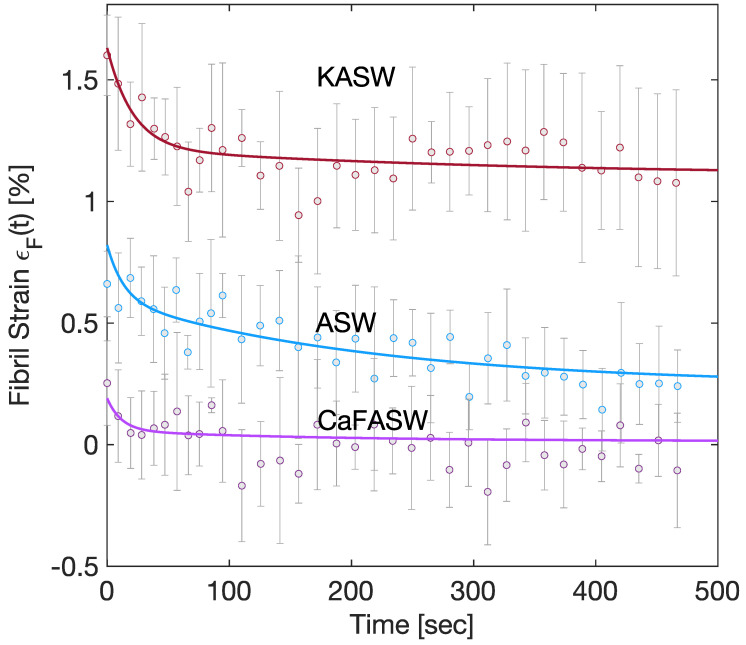
Chemoelastic relaxation for the fibril strain; the continuous lines are the numerical results, the dots are the mean of the experimental measures, and the error bar plots are the corresponding standard deviations.

Furthermore, experimentally, fibril strains are given simply by a one-term Prony series in the stiffened and softened state and by a two-term Prony series in the artificial seawater [8], as in Figure 3. From the approximate analytical solution in Appendix B,

(5) $$\epsilon_{F}\left(t\right)\approx \epsilon_{T}\;N\;\rho_{F}^{2}\;\sqrt{\phi /\pi }\;\frac{G_{m}\left(t\right)}{E_{F}\left(t\right)}$$

where $E_{F}$ is the fibril Young modulus.

In order to minimize the total number of parameters involved in the analysis, we assume that $E_{F}$ is expressed by a one-term Prony series:

(6) $$E_{F}\left(t\right)=E_{\infty }\;\left(1+e_{1}\;e^{-t/t_{K_{1}}}\right)$$

with $E_{\infty }$ being the asymptotic fibril Young modulus, $e_{1}$ being a dimensionless parameter of magnitude O(1), and $t_{K_{1}}$ a relaxation time constant.

From experimental values [8], we know that fibril strain decays rapidly, with a time constant close to $t_{M_{1}}$. As we can see from Equation (5), the ratio $G_{m}\left(t\right)/E_{F}\left(t\right)$ is close to a one-term Prony series with a time constant $t_{M_{1}}$ if $\gamma_{2}\approx e_{1}$ and if $t_{K_{1}}\approx t_{M_{2}}$, meaning that the time constant for the fibril chemoelastic relaxation must be close to the largest time constant for the interfibrillar matrix chemoelastic relaxation. We use these insights to guide the numerical optimizations to quantitatively obtain the parameters of the elastic models for the fibrils and matrix. Therefore, assuming

(7) $$G_{M}\left(t\right)=G_{\infty }\;+G_{1}\;e^{-t/t_{M_{1}}}+G_{2}\;e^{-t/t_{M_{2}}}\;E_{F}\left(t\right)=E_{\infty }+E_{1}\;e^{-t/t_{M_{2}}}$$

we aim to calculate $G_{\infty }$, $G_{1}$, $G_{2}$, $E_{\infty }$, and $E_{1}$, and we take $t_{M_{1}}$ and $t_{M_{2}}$ from the experimental values in [8].

We calculate the parameters $G_{\infty }$, $G_{1}$, $G_{2}$, $E_{\infty }$, and $E_{1}$ in Equation (7) through a least squares minimization of the difference between the stresses and strains from the finite element model and the experimental values.

In all the calculations, we assumed a tissue specimen length of $L_{T}=10\;\mathrm{mm}$, a fibril length of $L_{F}=100\;\mu m$, a fibril aspect ratio of $\rho_{F}=1000$, and an applied tissue strain of $\epsilon_{T}=50\%$. We used N=100 fibril elements and a final simulation time of 500s with a time step of Δt=1s.

We have two sets of experimental values for matching the model: tissue stress and the fibril strain. Because the tissue stress depends only on the matrix shear modulus, we can proceed in two stages. In the first one, we minimize the difference between the tissue stress values, obtaining, in this way, $G_{\infty }$, $G_{1}$, and $G_{2}$. With these parameters at hand, we move on to a second curve, fitting the fibril strains, where we get $E_{\infty }$ and $E_{1}$.

## 3. Results

Figure 2 and Figure 3 display the numerical results of such minimizations for the three seawater conditions, namely the Artificial (ASW, taken as reference solution), the potassium-enriched (KASW), and the calcium ions-deprived seawater (CaFASW).

**Figure 4:**
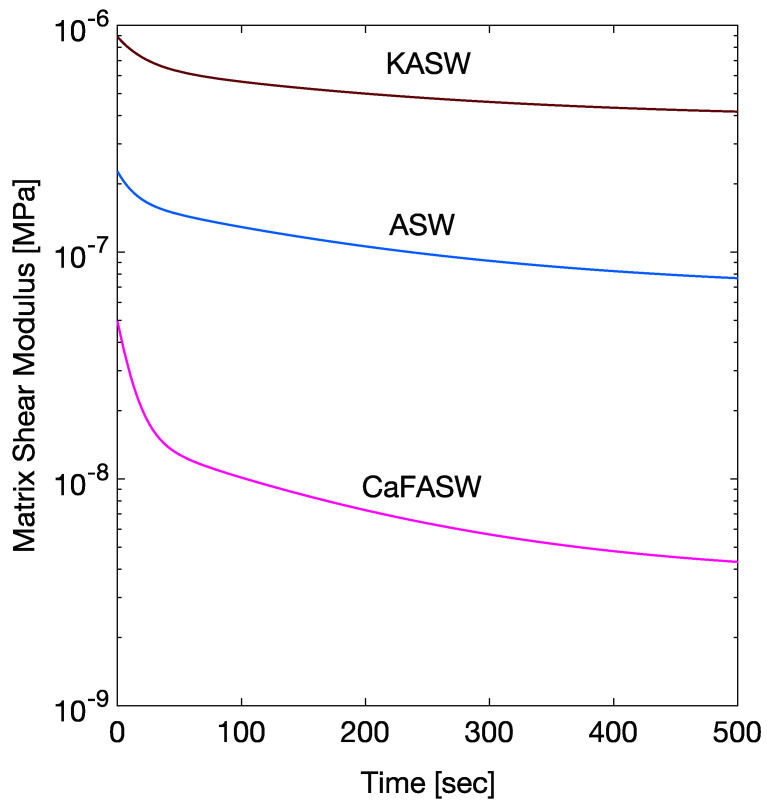
Chemoelastic relaxation parameters for the interfibrillar matrix.

**Figure 5:**
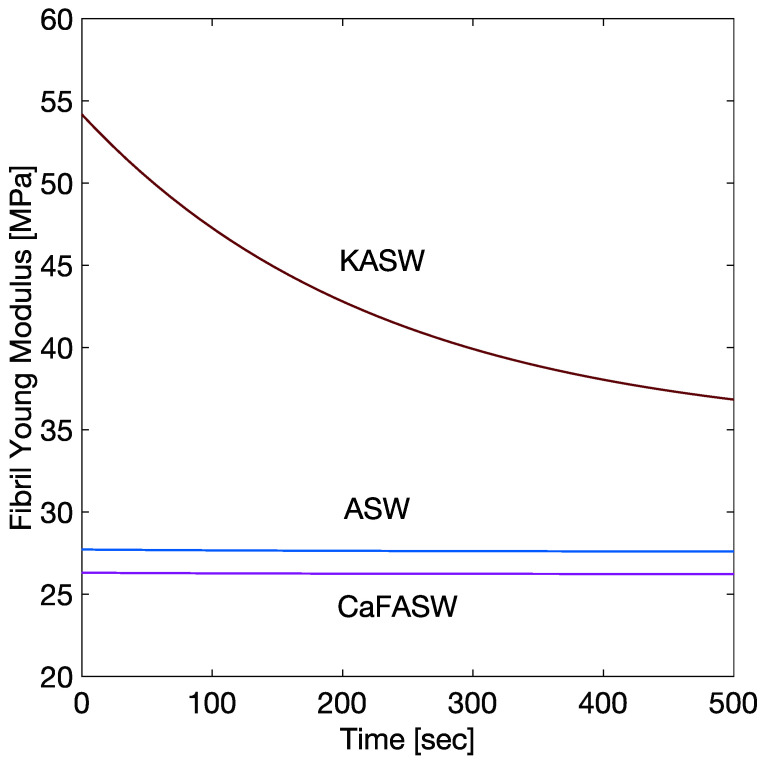
Chemoelastic relaxation parameters for the fibrils.

Table 1 summarizes the calculated chemoelastic parameters for the interfibrillar matrix ($G_{\infty }$, $G_{1}$, $G_{2}$) and for the fibrils ($E_{\infty }$, $E_{1}$, $t_{K_{1}}$). Figure 4 and Figure 5 show the corresponding time histories as in Equation (7). Figure 4 shows the softening in CaFASW and stiffening in KASW compared to ASW, which is of about an order of magnitude for the interfibrillar matrix. For the fibrils, as in Figure 5, in the KASW solution, the fibril relaxes chemoelastically, with a time constant equal to the longest tissue relaxation time. It is also about two times stiffer than the fibril in ASW and CaFASW. In contrast, the ASW and CaFASW solutions display no fibril chemical relaxation, with the fibrils in ASW slightly stiffer than in CaFASW.

## 4. Chemoelastic Parameters in Terms of Kinetic Reactions

We now attempt to explain these findings through kinetic reactions involving the scission and recombination of links [20,27].

**Figure 6:**
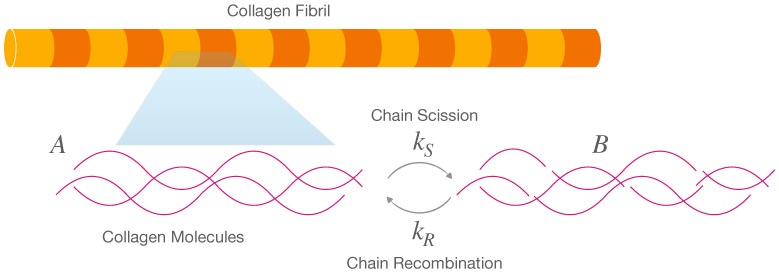
Elastic fibrils made by collagen macromolecules: hypothesized reversible reaction of chain scission and recombination; *A* are macro-chains before scission and *B* are those after scission.

For the fibrils, we consider the following reversible reaction, where the chain scission occurs in equilibrium with chain recombination (Figure 6):

(8) $$A\underset{k_{R}}{\overset{k_{S}}{⇌}}B$$

where *A* represents the macro-chains before scission, *B* represents the chains after scission, $k_{S}$ is the scission rate, and $k_{R}$ is the recombination rate. We assume that fibrils can only carry elastic loads if intact; therefore, the elastically active chains will depend on the concentration [A] of species *A*.

(9) $$E_{F}\left(t\right)=3\;\left[A\right]\;k\;T$$

where *k* is the Boltzmann constant, *T* is the absolute temperature, and the prefactor 3 appears because we assumed the fibril was incompressible.

**Figure 7:**
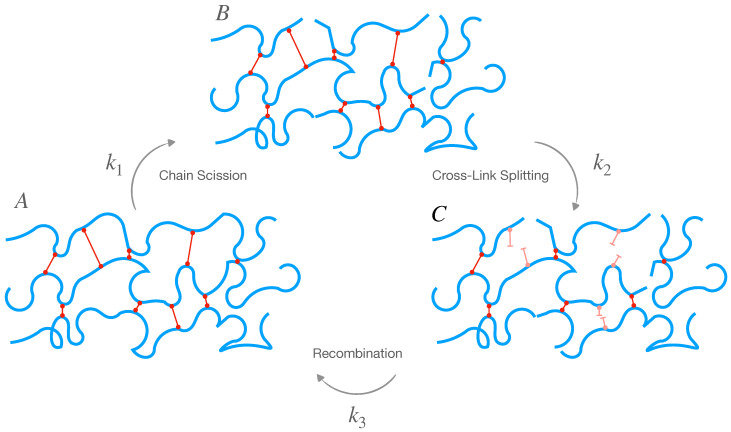
Interfibrillar matrix composed of crosslinked macro-chains (species **A**); the hypothesized reactions of chain scission, crosslink splitting, and recombination. Species **B** represents chains after chain scission and **C** is chains after chain scission and cross-link splitting.

For the interfibrillar matrix, we assume that two concurrent breakage mechanisms occur: the breaking of the intramolecular bond, namely chain scission, and the splitting of the crosslinks connecting different macro-chains (crosslink splitting). Additionally, we allow the possibility of simultaneous chain and crosslink recombination after these two splitting mechanisms (Figure 7). This picture is idealized, but it is a necessary simplification that introduces the least number of parameters (three reaction rates and three initial conditions) to fit the experimental data without too much indetermination.

(10) $$\begin{array}{c} A\overset{k_{1}}{⟶}B\\ B\overset{k_{2}}{⟶}C\\ C\overset{k_{3}}{⟶}A \end{array}$$

where *A* represents the crosslinked macro-molecular chains, *B* represents the macro-chains after chain scission (broken bond inside the chain) that are still crosslinked, *C* represents the macro-chains after chain scission and crosslink splitting, $k_{1}=1/t_{1}$ is the chain scission reaction rate ($t_{1}$ is a characteristic time), $k_{2}=1/t_{2}$ is the crosslink splitting reaction rate and $k_{3}=1/t_{3}$ is the recombination rate.

We assume the matrix loses elasticity if it is in configuration *C*. Therefore, the elastically active chains are those in configurations *A* and *B*; even if the chains are scissored, the matrix can still carry loads because the chains are crosslinked.

(11) $$G_{m}\left(t\right)=\left([A](t)+[B](t)\right)\;k\;T$$

where *k* is the Boltzmann constant, and *T* is the absolute temperature.

### 4.1. Fibril Chemoelasticity

Solution (A28) of the kinetic equation indicates that the sum of the scission rate and recombination rate is equal to the inverse of the time constant $t_{K_{1}}$, which, in all three chemical conditions, is close to the largest tissue stress relaxation time constant $=t_{M_{2}}$ (Table 1). The higher the initial percentage of undamaged collagen chains $a_{0}$, the higher the proportion of the recombination rate (A32). Additionally, the balance of the recombination rate depends on the ratio $E_{F_{\infty }}/\left(E_{F_{\infty }}+E_{1}\right)$. Table 1 and Figure 5 show that $E_{F}\left(t\right)$ is constant for ASW and CaFASW. This finding means that fibrils do not show chemoelasticity and are only elastic, which is compatible with the previous results [8,17]. Interestingly, in KASW, a chemoelastic relaxation is possible: the ratio $E_{F_{1}}/\left(E_{F_{\infty }}+E_{F_{1}}\right)$ is equal to 0.36. This means that for KASW

(12) $$\kappa_{R}=0.36\;a_{0}\;\kappa_{S}=1-0.36\;a_{0}$$

where $\kappa_{S}$ and $\kappa_{R}$ are the scission and recombination rates’ percentages. We are not able to infer $a_{0}$ from the experimental values. However, from Equation (12), we see that the recombination rate is always less than the scission rate, meaning that chain scission would occur much faster than chain recombination. The same applies to the interfibrillar matrix, as seen in the next subsection.

### 4.2. Matrix Chemoelasticity

**Figure 8:**
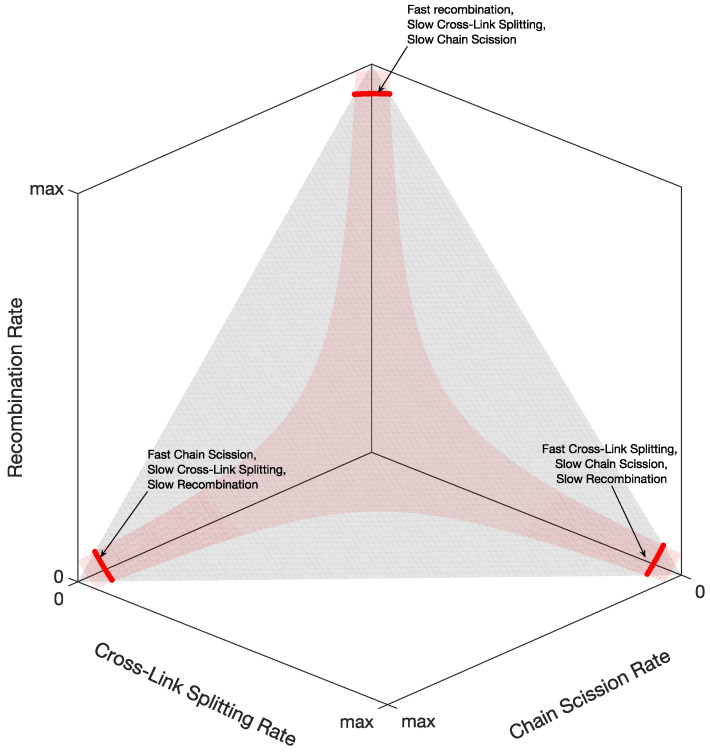
The surfaces in Equation (A47). The intersection of the surfaces (red lines) is all the possible reaction rates.

After solving the kinetic reaction Equation (A36) and comparing the solutions’ time constants with the tissue stress relaxation’s time constants, we obtain three possibilities for the reaction rates, as shown in Figure 8: (i) fast recombination, slow crosslink splitting, and slow chain scission; (ii) fast crosslink splitting, slow chain scission, and slow recombination; (iii) fast chain scission, slow crosslink splitting, and slow recombination.

However, considering the coefficients of the Prony series of the matrix shear modulus in Table 1, we found that only one of the three possibilities is feasible. Indeed, from the coefficients in Equation (7), we can calculate the percentages (Equation (A65)) of the initial reagents in Figure 7. It turns out that the only reaction rates giving physically relevant percentages (from 0 to 100) are slow recombination, fast crosslink splitting, and slow chain scission.

**Figure 9:**
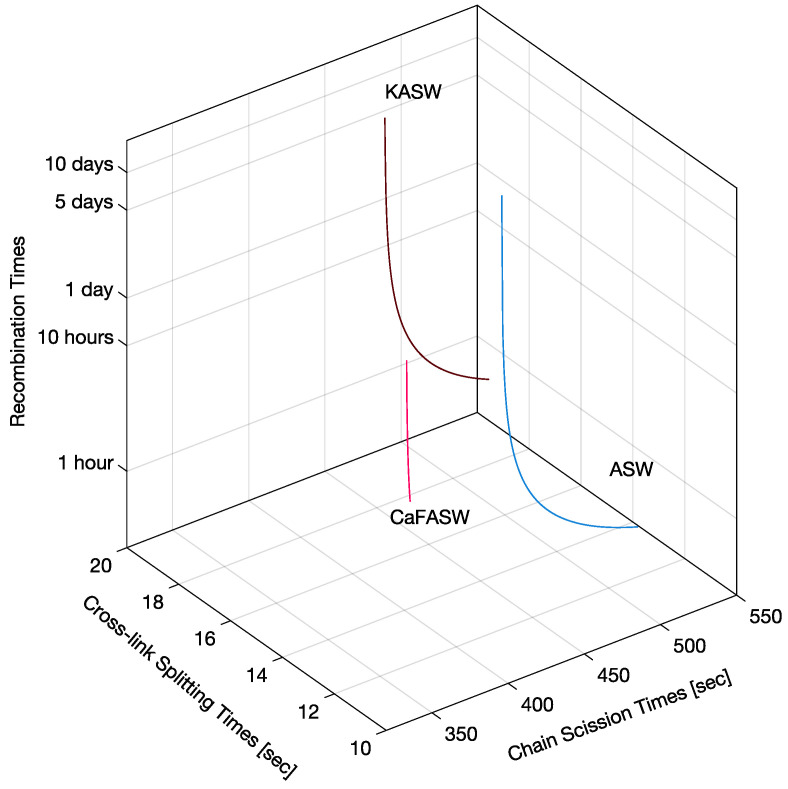
Interfibrillar matrix reaction times for ASW, KASW, and CaFASW: in all three conditions, the fastest times are due to chain recombination, with crosslink splitting being around 10 times slower and chain scission being extremely slow.

Figure 9 shows the corresponding reaction times, i.e., the inverse of the reaction rates from the thick red line on the right of Figure 8. We see that the recombination times are slow or extremely slow in the order of hours or days, whereas crosslink splitting is, at most, 20 s, and the chain scission times are, at most, 500 s (8 min). When comparing these reaction times with the matrix shear modulus time constants in Table 1, we see that the crosslink splitting times are similar to the smallest time constants, $t_{M_{1}}$, and the chain scission times are comparable to the largest time constants, $t_{M_{2}}$, for all three chemical environments. Additionally, we see that crosslink splitting and chain scission in CaFASW (softening solution) are faster than those in KASW (stiffening solution) and ASW (standard solution).

**Figure 10:**
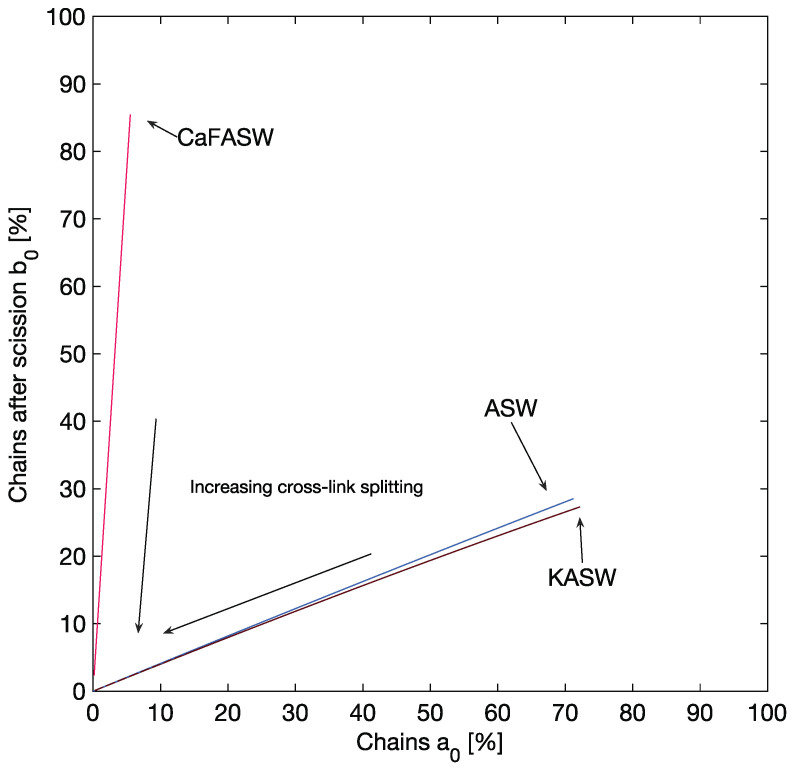
Initial conditions of the chemical species for all the permissible reaction times.

It is impossible to infer from the model and the experimental values in our possession the exact initial conditions of the reagents. However, we can compute the possible percentages $a_{0}$ and $b_{0}$ (A65). Figure 10 shows percentages $a_{0}$ (crosslinked macro-chains, *x*-axis) and $b_{0}$ (scissored chains, *y*-axis) for the given reaction rates: $k_{1}$ (chain scission), $k_{2}$ (crosslink splitting), and $k_{3}$ (recombination).

We notice that for ASW and KASW, the initial conditions are such that the intact chains percentage, $a_{0}$, is always larger than the ones, $b_{0}$, with internal damage, in a ratio of ${}_{0}/b_{0}=2.33$. This finding could lead to speculation that the crosslinking between chains increases in going from a standard to a stiff state.

A completely opposite trend is seen for CaFASW. The initial conditions are such that $a_{0}$ is low, less than 10%, meaning that, initially, most chains are damaged: either scissored ($b_{0}$) or with their crosslinks removed ($c_{0}=1-b_{0}-a_{0}$). This result confirms the intuition that the chains undergo significant scission in going from a standard state to a soft one.

## 5. Conclusions

We have hypothesized a relaxation mechanism for the mutable connective tissue of a sea cucumber. Our explanation is alternative to the commonly believed assumption that visco-elasticity is the only mechanism at play. Indeed, in our previous modeling work, we found some discrepancies when we framed the visco-elastic parameters in a polymer theory, such as the Doi-Edwards model.

We then turned to a completely different point of view. We used a chemoelastic framework, where the stiffness varies because of a change in the concentration of polymer macro-chains participating in the elastic response. Such change is described by first-order kinetic reactions.

These reactions involve cracking mechanisms. For the fibrils, the hypothesized mechanism is the scission of collagenous chains. For the matrix, we hypothesized two competing mechanisms: the chain scission and splitting of crosslinks. In both cases, we incorporated the possibility of chain and crosslink recombination.

Our mathematical model revealed that in standard chemical environments, such as ASW, and in softening conditions (CaFASW), fibrils are only elastic, with no stiffness change over time. Only in a stiffening seawater solution (KASW) did we find chemoelastic behavior. If the hypothesized mechanism is correct, such fibril stress relaxation occurs so that scission is always faster than recombination.

Finally, our model indicated that in all three chemical environments, the interfibrillar matrix relaxes primarily through crosslink splitting, where reaction times are in the order of 10 s, similar to the shortest tissue stress relaxation times measured in the experiments [8]. Chain scission participates more slowly, in the order of 5–10 min. Even slower is recombination, which can take place over hours for barely damaged macro-chains or days for highly damaged macro-chains.

A final finding led us to speculate on the transitions from standard to soft and standard to stiff. For instance, after computing the initial conditions compatible with the experiments, it could happen that the standard-to-stiff form occurs by increasing the crosslinks in the interfibrillar matrix and the recombination of collagen chains in the fibril; in contrast, the standard-to-soft form might occur only through chain scission. This is speculative because our model and experimental measures refer to stress relaxation in fixed rather than changing chemical environments, although it seems intuitive.

We would like to remark that we have proposed hypothetical mechanisms, albeit backed by mathematical modeling. Further experimental tests, for example, measuring stress while the water solution changes from standard to soft or standard to stiff, would provide additional insights for a more advanced mathematical model.

Such a sophisticated model could be, for example, a coupled multi-physics three-dimensional finite element model, where the ultra-structure is represented more accurately and more complex reaction kinetics are considered.

## Figures and Tables

**Table 1:** Chemoelastical parameters for the interfibrillar matrix shear modulus and fibril Young modulus for the three seawater solutions.

Seawater	$G_{\infty }$ (MPa)	$G_{1}$ (MPa)	$G_{2}$ (MPa)	$E_{\infty }$ (MPa)	$E_{1}$ (MPa)	$t_{M_{1}}$ (s) [8]	$t_{M_{2}}$ (s) [8]
ASW	6.6974$\cdot 10^{-8}$	6.1561$\cdot 10^{-8}$	9.8484$\cdot 10^{-8}$	27.59	0.1314	13.35	216
KASW	3.8535$\cdot 10^{-7}$	2.2968$\cdot 10^{-7}$	2.7412$\cdot 10^{-7}$	34.6294	19.5533	20.37	229.4
CaFASW	3.6746$\cdot 10^{-9}$	3.4478$\cdot 10^{-8}$	1.1543$\cdot 10^{-8}$	26.2296	0.0839	11.71	171.7

## Data Availability

The data presented in this study are available upon request from the corresponding author. The data are not publicly available due to privacy reasons.

## Abbreviations

The following abbreviations are used in this manuscript:

MCT	Mutable collagenous tissue
ECM	Extracellular matrix
JLC	Juxtaligamental cell
ASW	Artificial seawater
KASW	Potassium-enriched artificial seawater
CaFASW	Calcium ions-deprived artificial seawater

## Appendix A. Stiffness Matrices for Fibrillar MCT Matrix with Visco- and Chemoelasticity

### Appendix A.1. Finite Element Model

To derive the stiffness matrix of the fibrillar MCT matrix, we construct the following model composed of one-dimensional, staggered finite elements.

With reference to Figure 1, in such a finite element model, the displacements u are known only at the extremities of the fibrils (the nodes). The nodes are numbered from 1 to *n* (Figure 1b), and the displacements are grouped in a vector

(A1) $$u=\left(\begin{array}{c} u_{1}\\ u_{2}\\ \ldots \\ u_{n} \end{array}\right)$$

Furthermore, our finite element model assumes that displacements vary linearly along the fibril; therefore, the strain inside each fibril is uniform.

In a finite element model, the unknown displacements u are obtained from the algebraic system of equations

(A2) Ku=F

where K is the stiffness matrix, and F is the external forces vector. Matrix K comes from the internal forces derived from the material reaction to the external forces.

We apply opposite displacements $\epsilon_{T}\;L_{T}$ at nodes 1 and *n*, with, $\epsilon_{T}$, the applied tissue strain, and, $L_{T}$, the length of the tissue sample:

(A3) $$v=\left(\begin{array}{c} u_{1}\\ u_{n} \end{array}\right)=\frac{1}{2}\;\epsilon_{T}\;L_{T}\;\left(\begin{array}{c} -1\\ 1 \end{array}\right)$$

Equation (A2) can be rearranged as

(A4) $$\left(\begin{array}{cc} K_{vv} & K_{vx}\\ {K_{vx}}^{T} & K_{xx} \end{array}\right)\left(\begin{array}{c} v\\ x \end{array}\right)=\left(\begin{array}{c} R\\ P \end{array}\right)$$

with R being the reaction force and x the unknown fibril displacements. Equation (A4) can be solved

(A5) $$x={K_{xx}}^{-1}\;\left(P-{K_{vx}}^{T}\;v\right)$$

and the reaction forces computed from the known macroscopic $\epsilon_{T}$ are

(A6) $$R=K_{vv}\;v+K_{vx}\;x$$

Vector R has two components, and $R_{1}=-R_{2}$.

The tissue stress $\sigma_{T}$ can be computed as

(A7) $$\sigma_{T}=\frac{\rho_{F}^{2}}{L_{F}^{2}}\;R_{2}$$

where $L_{F}$ is the fibril length, and $\rho_{F}$ is the fibril aspect ratio, i.e., $\rho_{F}=L_{F}/r_{F}$, where $r_{F}$ is the radius of the fibril’s cross-section, which is assumed to be cylindrical.

The fibril strain $\epsilon_{F}$ can be computed from the displacements u, for example, for the first fibril with nodes 1 and 2:

(A8) $$\epsilon_{F}\left(t\right)=\frac{u_{2}\left(t\right)-u_{1}\left(t\right)}{L_{F}}$$

### Appendix A.2. Staggered Shear-Lag Model

Because of the symmetry, we will focus only on two rows of such elements (Figure 1a). The horizontal rows represent fibrils, which, for simplicity, we assume to be a cylindrical shape, with a stiffness of $k=\pi \;E_{F}\;{r_{F}}^{2}/L_{F}$, with $E_{F}$ being the fibril Young modulus. The rows are connected transversely by shear elements (Figure 1b). For example, two shear stresses act on node 3 of elements 3−4, namely $\tau_{13}$ and $-\tau_{35}$, whereas on node 4, the shear stresses are $\tau_{24}$ and $-\tau_{46}$ (Figure 1c), with

(A9) $$\tau_{ij}=G_{m}\;\frac{u_{j}-u_{i}}{h}$$

where $(u_{j}-u_{i})/h$ is the approximated shear strain when $h\ll L_{F}$, $u_{i}$ the horizontal displacement of node *i*, $G_{m}$ is the interfibrillar matrix shear modulus, *h* the separation between the two rows. According to this scheme, the resulting equations of equilibrium of forces on elements 3−4 are

(A10) $$\left(\begin{array}{cc} k & -k\\ -k & k \end{array}\right)\;\left(\begin{array}{c} u_{3}\\ u_{4} \end{array}\right)+\left(\begin{array}{c} m\;\left(u_{3}-u_{1}\right)\\ m\;\left(u_{4}-u_{2}\right) \end{array}\right)-\left(\begin{array}{c} m\;\left(u_{5}-u_{3}\right)\\ m\;\left(u_{6}-u_{4}\right) \end{array}\right)=\left(\begin{array}{c} 0\\ 0 \end{array}\right)$$

with $m=2\;\pi \;r_{F}\;L_{F}\;\frac{G_{m}}{h}$.

We can rewrite Equation (A10) as a matrix-vector product:

(A11) $$k\;\left(\begin{array}{cccccc} 0 & 0 & 1 & -1 & 0 & 0\\ 0 & 0 & -1 & 1 & 0 & 0 \end{array}\right)\;\left(\begin{array}{c} u_{1}\\ u_{2}\\ u_{3}\\ u_{4}\\ u_{5}\\ u_{6} \end{array}\right)+m\;\left(\begin{array}{cccccc} -1 & 0 & 2 & 0 & -1 & 0\\ 0 & -1 & 0 & 2 & 0 & -1 \end{array}\right)\;\left(\begin{array}{c} u_{1}\\ u_{2}\\ u_{3}\\ u_{4}\\ u_{5}\\ u_{6} \end{array}\right)=\left(\begin{array}{c} 0\\ 0 \end{array}\right)$$

Let us consider a finite element model with *n* nodes, with *n* being an even number; the number of elements is then n/2. A staggered shear-lag model’s global stiffness matrix K follows the pattern in Equation (A11). When taking into account all the fibrils, the K ends up being the sum of two contributions

(A12) $$K\left(t\right)=k\left(t\right)\;A^{E}+m\left(t\right)\;A^{S}$$

with $A^{E}$ being the assembly matrix for the elastic elements and $A^{S}$ the assembly matrix for the shear elements. The matrix $A^{E}$ is a block diagonal matrix:

(A13) $$A^{E}=\left(\begin{array}{ccccc} A & \; & & \; & \\ \; & A & \; & & \\ \; & \; & A & \; & \\ \; & \; & & \ldots & \\ \; & \; & & \; & A \end{array}\right)\;A=\left(\begin{array}{cc} 1 & -1\\ -1 & 1 \end{array}\right)$$

The matrix $A^{S}$ is a tridiagonal block matrix:

(A14) $$A^{S}=\left[\begin{array}{cccc} I & -I & \; & 0\\ -I & 2\;I & -I & \\ \; & \ddots & \ddots & \ddots \\ 0 & \; & -I & I \end{array}\right]$$

with I being a 2×2 identity matrix.

## Appendix B. Approximate Analytical Solution for the Case Chemoelastic Matrix and Chemoelastic Fibril

Equation (A5) can be solved analytically for a system with relatively few elements. The number of elements we choose is 9 because, with more elements, the analytical inversion of the stiffness matrix becomes impractical and, therefore, must be solved numerically.

A close inspection of the strains revealed a characteristic feature of the solution for the shear-lag model: the fibrils experiencing the largest strain are the ones where the load is applied. Then, the strains decay rapidly from the application point. The ones in the middle are, therefore, approximately rigid.

The maximum fibril strain is

(A15) $$\frac{\epsilon_{F}\left(t\right)\;L_{F}}{\epsilon_{T}\;L_{T}}=\frac{\left(8\;m\right)\;k^{4}+\left(32\;m^{2}\right)\;k^{3}+\left(40\;m^{3}\right)\;k^{2}+\left(16\;m^{4}\right)\;k+m^{5}}{64\;k^{5}+\left(296\;m\right)\;k^{4}+\left(464\;m^{2}\right)\;k^{3}+\left(280\;m^{3}\right)\;k^{2}+\left(52\;m^{4}\right)\;k+m^{5}}$$

and the tissue stress is

(A16) $$\sigma_{T}\left(t\right)=\epsilon_{T}\;L_{T}\;\frac{\rho_{F}^{2}}{\pi \;L_{F}^{2}}\;\frac{\left(16\;m\right)\;k^{5}+\left(72\;m^{2}\right)\;k^{4}+\left(108\;m^{3}\right)\;k^{3}+\left(60\;m^{4}\right)\;k^{2}+\left(9\;m^{5}\right)\;k}{64\;k^{5}+\left(296\;m\right)\;k^{4}+\left(464\;m^{2}\right)\;k^{3}+\left(280\;m^{3}\right)\;k^{2}+\left(52\;m^{4}\right)\;k+m^{5}}$$

and, since m/K≪1, their approximations are

(A17) $$\frac{\epsilon_{F}\left(t\right)\;L_{F}}{\epsilon_{T}\;L_{T}}\approx \frac{1}{8}\;\frac{m(t)}{k(t)}$$

(A18) $$\sigma_{T}\left(t\right)\approx \frac{1}{4}\;\epsilon_{T}\;L_{T}\;\frac{\rho_{F}^{2}}{\pi \;L_{F}^{2}}\;m\left(t\right)$$

Finally, by substituting for expressions m(t) and k(t) and considering that, approximately, $L_{T}/L_{F}\approx N$ is the number of fibrils in the sample, we obtain

(A19) $$\epsilon_{F}\left(t\right)\approx N\;\rho_{F}^{2}\;\sqrt{\phi /\pi }\;\frac{G_{m}\left(t\right)}{E_{F}\left(t\right)}\;\epsilon_{T}$$

and

(A20) $$\sigma_{T}\left(t\right)\approx \frac{1}{2}\;N\;\rho_{F}^{2}\;\sqrt{\phi /\pi }\;G_{m}\left(t\right)\;\epsilon_{T}\approx E_{F}\left(t\right)\;\epsilon_{F}\left(t\right)$$

where $\rho_{F}$ is the fibril aspect ratio, and ϕ is the fibril volume fraction.

## Appendix C. Elastically Active Chains after Chain Scission and Recombination

Assuming that the collagen fibril behaves like an incompressible linear elastic solid under an applied strain, $\epsilon_{F}$, the stress in the fibril is

(A21) $$\sigma_{F}\left(0\right)=E_{F}\left(0\right)\;\epsilon_{F}=3\;G_{F}\left(0\right)\;\epsilon_{F}$$

where $G_{F}$ is the fibril shear modulus. According to the kinetic theory of rubber elasticity [21], the shear modulus is given by

(A22) $$G_{F}=\left[A\right]\;k\;T$$

where [A] is the number density of elastically active chains (chains supporting the tensile stress per unit volume), *k* is the Boltzmann constant, and *T* is the absolute temperature.

According to [20], chemical stress relaxation occurs because of a change in [A]:

(A23) $$\sigma_{F}\left(t\right)=3\;\left[A\right]\left(t\right)\;k\;T\;\epsilon_{F}$$

Therefore, using $\epsilon_{F}$ from Equation (A21), we obtain

(A24) $$\frac{\sigma_{F}\left(t\right)}{\sigma_{F}\left(0\right)}=\frac{E_{F}\left(t\right)}{E_{F}\left(0\right)}=\frac{\left[A](t\right)}{\left[A](0\right)}$$

The kinetic equation associated with Reaction (8) is

(A25) $$\frac{d[A]}{dt}=-k_{S}\left[A\right]+k_{R}\;\left[B\right]$$

For the mass balance

(A26) [A](0)+[B](0)=[A](t)+[B](t)

By substituting Equation (A26) into Equation (A25) and introducing the dimensionless variable a=[A]/([A](0)+[B](0)), we obtain

(A27) $$\frac{da}{dt}=-\left(k_{R}+k_{S}\right)\;b+k_{R}$$

The solution to Equation (A27) with initial condition $a_{0}$ is

(A28) $$a\left(t\right)=\left(a_{0}-a_{\infty }\right)\;e^{-(k_{S}+k_{R})\;t}+a_{\infty }$$

with $a_{\infty }$ being the asymptotic dimensionless number density

(A29) $$a_{\infty }=\frac{k_{R}}{k_{S}+k_{R}}$$

The time constant $k_{S}+k_{R}$ is the inverse of the time constant $t_{K_{1}}$ in Equation (6):

(A30) $$k_{S}+k_{R}=k_{K_{1}}=\frac{1}{t_{K_{1}}}$$

Hence, recalling Equation (A24),

(A31) $$\frac{E_{F_{\infty }}}{E_{F}\left(0\right)}=\frac{{\left[A\right]}_{\infty }}{A(0)}=\frac{a_{\infty }}{a_{0}}=\frac{1}{a_{0}}\;\frac{k_{R}}{k_{S}+k_{R}}$$

Let us normalize the reaction rates as $\kappa_{S}=k_{S}/k_{K_{1}}$ and $\kappa_{R}=k_{R}/k_{K_{1}}$. Finally,

(A32) $$\kappa_{R}=\frac{E_{F_{\infty }}}{E_{F_{\infty }}+E_{1}}\;a_{0}\;\kappa_{S}=1-\frac{E_{F_{\infty }}}{E_{F_{\infty }}+E_{1}}\;a_{0}$$

## Appendix D. Elastically Active Chains after Chain Scission, Cross-Links Splitting, and Chain Recombination

Assuming that the interfibrillar matrix can withstand only shear stresses and behaves like an incompressible Neo-Hookean solid, then, under a shear strain γ, the shear stress τ is

(A33) $$\tau \left(0\right)=G_{m}\left(0\right)\;\gamma$$

For the rubber theory of elasticity [21],

(A34) $$G_{m}\left(t\right)=\left([A](t)+[B](t)\right)\;k\;T$$

where [A](t)+[B](t) is the number density of elastically active chains (chains supporting the stress per unit volume), *k* is the Boltzmann constant, and *T* is the absolute temperature. Similarly to Appendix C, then

(A35) $$\frac{G_{m}\left(t\right)}{G_{m}\left(0\right)}=\frac{\left[A](t)+[B](t)(t\right)}{\left[A](0)+[B](0\right)}$$

The kinetic equations for Reaction (10) are

(A36) $$\begin{array}{c} \frac{d[A]}{dt}=k_{3}\;\left[C\right]-k_{1}\;\left[A\right]\\ \frac{d[B]}{dt}=k_{1}\;\left[A\right]-k_{2}\;\left[B\right]\\ \frac{d[C]}{dt}=k_{2}\;\left[B\right]-k_{3}\;\left[C\right] \end{array}$$

where [·] is the number density of the reacting species. Equation (A36) is not independent: summing the equations leads to the conservation of chemical species

(A37) $$\frac{d[A]}{dt}+\frac{d[B]}{dt}+\frac{d[C]}{dt}=0$$

However, we can write

(A38) $$\left[C\right]={\left[A\right]}_{0}+{\left[B\right]}_{0}+{\left[C\right]}_{0}-\left[A\right]-\left[B\right]$$

with ${\left[\cdot \right]}_{0}$ being the initial concentrations. By substituting Equation (A38) into Equation (A36), and rewriting in matrix form, we obtain

(A39) $$\begin{array}{c} \dot{Y}=K\;Y+B\\ Y\left(0\right)=Y_{0} \end{array}$$

with

(A40) $$K=\left(\begin{array}{cc} -(k_{1}+k_{3}) & -k_{3}\\ k_{1} & -k_{2} \end{array}\right)\;\;\;B=k_{3}\;\left({\left[A\right]}_{0}+{\left[B\right]}_{0}+{\left[C\right]}_{0}\right)\;\left(\begin{array}{c} 1\\ 0 \end{array}\right)\;\;\;Y\left(t\right)=\left(\begin{array}{c} \left[A(t)\right]\\ \left[B(t)\right] \end{array}\right)\;\;\;Y_{0}=\left(\begin{array}{c} {}[A{]}_{0}\\ {}[B{]}_{0} \end{array}\right)$$

We can introduce a dimensionless form:

(A41) $$y=\frac{1}{{\left[A\right]}_{0}+{\left[B\right]}_{0}+{\left[C\right]}_{0}}\;Y\;y=\left(\begin{array}{c} a(t)\\ b(t) \end{array}\right)\;b=k_{3}\;\left(\begin{array}{c} 1\\ 0 \end{array}\right)$$

The first-order system of ODEs (A39) has the following solution:

(A42) $$y\left(t\right)=e^{K\;t}\;\left(y_{0}-y_{b}\right)+y_{b}\;y_{b}=-K^{-1}\;b$$

and

(A43) $$e^{K\;t}=U\;e^{D\;t}\;U^{-1}$$

where D is a diagonal matrix with the eigenvalues of K, and U is the matrix for which the columns are the eigenvectors of K.

(A44) $$D=-\frac{1}{2}\;\left(\begin{array}{cc} k_{1}+k_{2}+k_{3}+\sigma & 0\\ 0 & k_{1}+k_{2}+k_{3}-\sigma \end{array}\right)\;\;\;U=\left(\begin{array}{cc} -\frac{k_{1}+k_{2}-k_{3}+\sigma }{2\;k_{2}} & -\frac{k_{1}+k_{2}-k_{3}-\sigma }{2\;k_{2}}\\ 1 & 1 \end{array}\right)$$

and

(A45) $$\sigma =\sqrt{{k_{1}}^{2}+{k_{2}}^{2}+{k_{3}}^{2}-2\;\left(k_{1}\;k_{2}+k_{2}\;k_{3}+k_{1}\;k_{3}\right)}$$

The eigenvalues of K are the inverse of the two time constants:

$$\frac{1}{2}\;\left(k_{1}+k_{2}+k_{3}-\sqrt{{k_{1}}^{2}+{k_{2}}^{2}+{k_{3}}^{2}-2\;\left(k_{1}\;k_{2}+k_{2}\;k_{3}+k_{1}\;k_{3}\right)}\right)=k_{M_{2}}=\frac{1}{t_{M_{2}}}$$

(A46) $$\frac{1}{2}\;\left(k_{1}+k_{2}+k_{3}+\sqrt{{k_{1}}^{2}+{k_{2}}^{2}+{k_{3}}^{2}-2\;\left(k_{1}\;k_{2}+k_{2}\;k_{3}+k_{1}\;k_{3}\right)}\right)=k_{M_{1}}=\frac{1}{t_{M_{1}}}$$

Manipulating Equation (A46) leads to the following:

(A47) $$\begin{array}{c} k_{1}+k_{2}+k_{3}=k_{M_{1}}+k_{M_{2}}\\ k_{1}\;k_{3}+k_{2}\;k_{3}+k_{1}\;k_{2}=k_{M_{1}}\;k_{M_{2}} \end{array}$$

The intersection of the two surfaces in Equation (A47) represents the possible reaction rates (Figure 8).

We can normalize the reaction rates $k_{i}\;i=1,2,3$

(A48) $$\kappa_{i}=\frac{k_{i}}{k_{M_{1}}+k_{M_{2}}}\;i=1,2,3$$

and combine Equation (A47) to get

(A49) $$\kappa_{1}+\kappa_{2}-\left({\kappa_{1}}^{2}+{\kappa_{2}}^{2}\right)=\frac{k_{M_{1}}\;k_{M_{2}}}{{\left(k_{M_{1}}+k_{M_{2}}\right)}^{2}}\;\kappa_{1}+\kappa_{2}\leq 1$$

Equation (A49) can be solved numerically, leading to all possible solutions $\kappa_{1}$ and $\kappa_{2}$. One way of solving it is to plot Equation (A49) as an implicit curve in the plane $\left(\kappa_{1},\kappa_{2}\right)$ and then extract its co-ordinates. Not all these rates are physical solutions; only those leading to $a_{0}$ (unbroken chains), $b_{0}$ (chains after internal scission), and $c_{0}=1-a_{0}-b_{0}$ (chains after scission and crosslink splitting) between 0 and 1 are acceptable solutions. With these values $\left(\kappa_{1},\kappa_{2}\right)$ at hand, and with $\kappa_{3}=1-\kappa_{1}-\kappa_{2}$, we can deduce the initial percentages as follows.

Let us examine the solutions a(t) and b(t).

(A50) $$a\left(t\right)=a_{\infty }+a^{\left(1\right)}\;e^{-t/t_{M_{1}}}+a^{\left(2\right)}\;e^{-t/t_{M_{2}}}$$

with

(A51) $$a_{\infty }=\kappa_{2}\;\kappa_{3}\;\frac{{\left(k_{M_{1}}+k_{M_{2}}\right)}^{2}}{k_{M_{1}}\;k_{M_{2}}}$$

(A52) $$\alpha =\frac{k_{M_{1}}+k_{M_{2}}}{k_{M_{1}}-k_{M_{2}}}$$

(A53) $$a^{\left(1\right)}=\frac{1}{2}\;\left(\alpha \;\left(1-2\;\kappa_{2}\right)+1\right)\;a_{0}+\frac{1}{4\;\alpha \;\kappa_{1}}\;\left(\alpha^{2}\;{\left(1-2\;\kappa_{2}\right)}^{2}-1\right)\;b_{0}+$$

$$-a_{\infty }\left(\;\frac{1}{2}\;\left(\alpha \;\left(1-2\;\kappa_{2}\right)+1\right)+\frac{1}{4\;\alpha \;\kappa_{2}}\left(\alpha^{2}\;{\left(1-2\;\kappa_{2}\right)}^{2}-1\right)\;\right)=A_{11}\;a_{0}+A_{12}\;b_{0}+A_{13}\;a_{\infty }$$

(A54) $$a^{\left(2\right)}=\frac{1}{2}\;\left(1-\alpha \;\left(1-2\;\kappa_{2}\right)\right)\;a_{0}+\frac{1}{4\;\alpha \;\kappa_{1}}\;\left(1-\alpha^{2}\;{\left(1-2\;\kappa_{2}\right)}^{2}\right)\;b_{0}+$$

$$+a_{\infty }\left(\;\frac{1}{2}\;\left(-1+\alpha \;\left(1-2\;\kappa_{2}\right)\right)+\frac{1}{4\;\alpha \;\kappa_{2}}\;\left(\alpha^{2}\;\left(1-2\;\kappa_{2}\right)-1\right)\;\right)=A_{21}\;a_{0}-A_{12}\;b_{0}+A_{23}\;a_{\infty }$$

It can be verified that

(A55) $$a\left(0\right)=a^{\left(1\right)}+a^{\left(2\right)}+a_{\infty }=a_{0}$$

Moreover,

(A56) $$b\left(t\right)=b_{\infty }+b^{\left(1\right)}\;e^{-t/t_{M_{1}}}+b^{\left(2\right)}\;e^{-t/t_{M_{2}}}$$

(A57) $$b_{\infty }=\kappa_{1}\;\kappa_{3}\;\frac{{\left(k_{M_{1}}+k_{M_{2}}\right)}^{2}}{k_{M_{1}}\;k_{M_{2}}}$$

(A58) $$b^{\left(1\right)}=-\alpha \;\kappa_{1}\;a_{0}+\frac{1}{2}\;\left(1-\alpha \;\left(1-2\;\kappa_{2}\right)\right)\;b_{0}+\frac{1}{2}\;\left(\alpha -1\right)\;b_{\infty }=B_{11}\;a_{0}+B_{12}\;b_{0}+B_{13}\;b_{\infty }$$

(A59) $$b^{\left(2\right)}=\alpha \;\kappa_{1}\;a_{0}+\frac{1}{2}\;\left(1+\alpha \;\left(1-2\;\kappa_{2}\right)\right)\;b_{0}-\frac{1}{2}\;\left(\alpha +1\right)\;b_{\infty }=-B_{11}\;a_{0}+B_{21}\;b_{0}+B_{23}\;b_{\infty }$$

it can be verified that

(A60) $$b\left(0\right)=b^{\left(1\right)}+b^{\left(2\right)}+b_{\infty }=b_{0}$$

To compute $a_{0}$ and $b_{0}$, we set the Prony series coefficients for $\left(a\left(t\right)+b\left(t\right)\right)/\left(a_{0}+b_{0}\right)$ to be equal to those of $G_{M}\left(t\right)/G_{M}\left(0\right)$.

(A61) $$G_{M}\left(t\right)/G_{M}\left(0\right)=g_{\infty }+g_{1}\;e^{-\frac{t}{t_{M_{1}}}}+g_{2}\;e^{-\frac{t}{t_{M_{2}}}}$$

with

(A62) $$g_{\infty }=\frac{G_{\infty }}{G_{\infty }+G_{1}+G_{2}}\;g_{1}=\frac{G_{1}}{G_{\infty }+G_{1}+G_{2}}\;g_{2}=\frac{G_{2}}{G_{\infty }+G_{1}+G_{2}}$$

From Solution (A42), we obtain

(A63) $$a\left(t\right)+b\left(t\right)=\left(a_{\infty }+b_{\infty }\right)+\left(a^{\left(1\right)}+b^{\left(1\right)}\right)\;e^{-\frac{t}{t_{M_{1}}}}+\left(a^{\left(2\right)}+b^{\left(2\right)}\right)\;e^{-\frac{t}{t_{M_{2}}}}$$

Since $g_{\infty }=1-g_{1}-g_{2}$, we only need to set two conditions on the coefficients. Therefore, we pose

(A64) $$\left(a^{\left(1\right)}+b^{\left(1\right)}\right)-\left(a_{0}+b_{0}\right)\;g_{1}=0\;\left(a^{\left(2\right)}+b^{\left(2\right)}\right)-\left(a_{0}+b_{0}\right)\;g_{2}=0$$

Substituting Equations (A53), (A54), (A58), and (A59) into Equation (A64) results in the linear system of equations:

(A65) $$C\;\left(\begin{array}{c} a_{0}\\ b_{0} \end{array}\right)=d$$

with

(A66) $$C=\left(\begin{array}{cc} A_{11}+B_{11}-g_{1} & A_{12}+B_{12}-g_{1}\\ A_{21}-B_{11}-g_{2} & -A_{12}+B_{21}-g_{2} \end{array}\right)$$

(A67) $$d=-\left(\begin{array}{c} A_{13}\;a_{\infty }+B_{13}\;b_{\infty }\\ A_{23}\;a_{\infty }+B_{23}\;b_{\infty } \end{array}\right)$$

Therefore,

(A68) $$\left(\begin{array}{c} a_{0}\\ b_{0} \end{array}\right)=C^{-1}\;d$$

We select only $\kappa_{1}$, $\kappa_{2}$, $\kappa_{3}$, $a_{0}$, $b_{0}$, and $c_{0}=1-a_{0}-b_{0}$ such that $0\leq a_{0}\leq 1$, $0\leq b_{0}\leq 1$, and $0\leq c_{0}\leq 1$.
